# Human sciences can increase technology acceptance in rehabilitation science: a call for action

**DOI:** 10.3389/fresc.2024.1356445

**Published:** 2025-01-30

**Authors:** Matthias Ertl, Lilla M. Gurtner

**Affiliations:** ^1^ Institute for Psychology, University of Bern, Bern, Switzerland; ^2^ Neurocenter, Luzerner Kantonsspital, Lucerne, Switzerland; ^3^ Faculty of Behavioural Sciences and Psychology, University of Lucerne, Lucerne, Switzerland; ^4^ Centre for Development and Environment, University of Bern, Bern, Switzerland

**Keywords:** rehabilitation, demographic transition, technology acceptance, behaviour change, interdisciplinarity, psychology, labor shortage, healthcare

## A changing world

**Figure 1:**
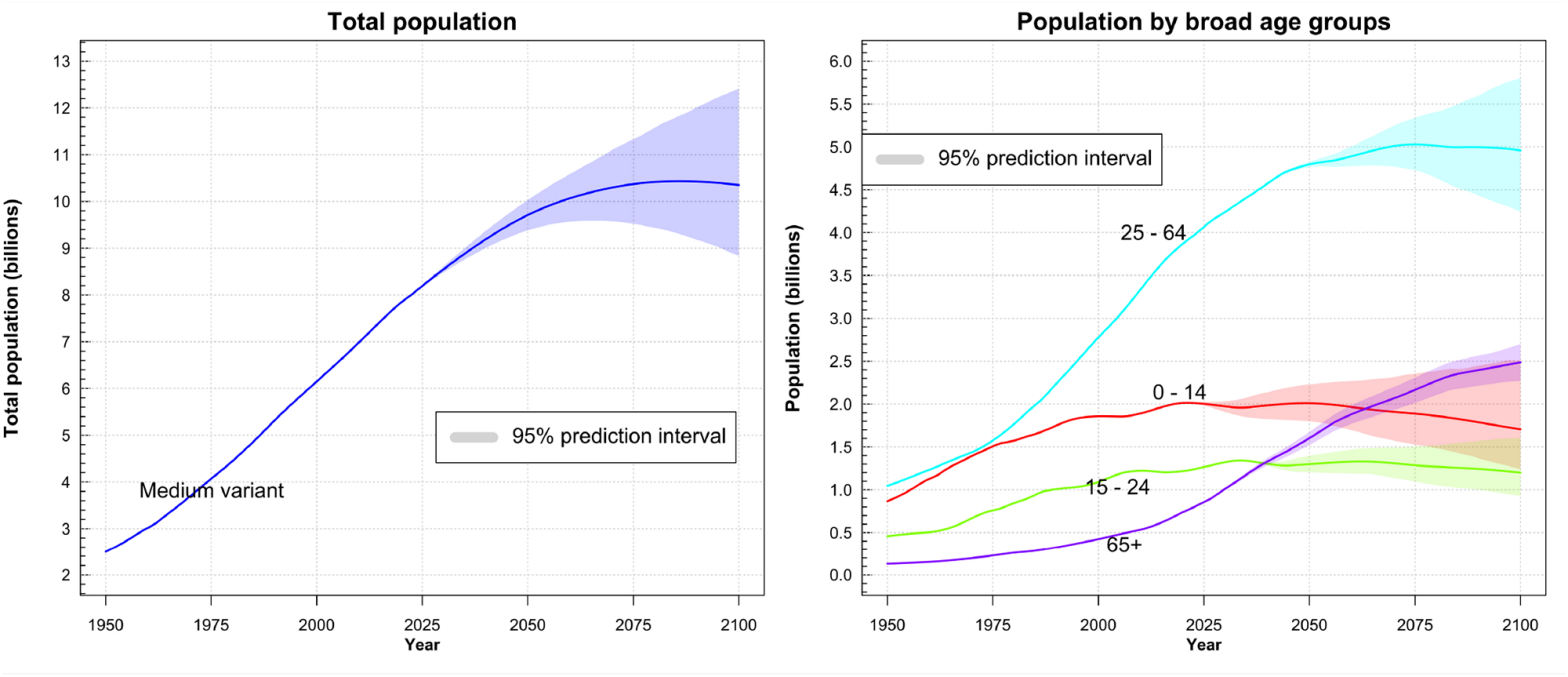
**Left:** Visualization of the recorded and predicted world population. The purple area represents the projected outcome based on varying conditions in the model. The median variant suggests a maximum world population of 10.5 billion people around the year 2080. **Right:** Recorded and predicted population growth among various age groups. The models suggest a doubling of older people (65+ years) with in the next 25 years. ©2022 United Nations, DESA, Population Division. Licensed under Creative Commons license CC BY 3.0 IGO. United Nations, DESA, Population Division. World Population Prospects 2022. http://population.un.org/wpp/.

According to the *World Population Prospects 2022* report by the United Nations (1), the global population growth is projected to decelerate in the coming decades, potentially reaching its zenith by the close of the century. However, the demographic cohort comprising individuals aged 65 years and above is rapidly expanding and will continue to do so in the ensuing decades (see Figure 1). Just until the mid of the 21st century, the share of this demographic is anticipated to surge from 18.7% (in 2022) to 26.9% (in 2050) in Europe and Northern America. This demographic transition impacts the labor market, pension systems, and notably, the healthcare infrastructure.

The repercussions for healthcare systems stem from two primary factors. First, an aging population means increasing prevalence of various disorders and conditions (e.g., Alzheimer’s disease, stroke), as well as impairments (e.g., hearing loss) in need of treatment. In fact, a recent, global study (2) estimated that the number of people potentially in need of rehabilitation services already increased by 63% between 1990 and 2019, due to a growing and aging world population. Second, this escalating demand is set to collide with a shortage of healthcare professionals. The World Health Organization (WHO) foresees a deficit of 10 million health workers by 2030, impacting nations across all socioeconomic development levels, with a particularly bleak outlook for low- and lower-middle-income countries (3). Global inequalities already cause significant migration of health care workers from the Global South to the Global North, leaving the health care work force in the originating countries depleted (4). This confluence of heightened demand combined with a diminishing workforce poses a considerable challenge for healthcare systems (which are already contending with personnel shortages, low wages and high demands in flexibility, responsibility and physical and mental burden). If not prevented, it leads to a reduction in service provision and/or quality, which increases human suffering.

Although the details may vary with respect to countries and regions, it is evident that many societies worldwide will face an aging population combined with a contracting workforce in the forthcoming decades, exerting significant ramifications on the healthcare sector. Consequently, there is an imperative need to formulate and implement mitigation strategies addressing this challenge across various domains, including rehabilitation.

## Is technological innovation the solution?

To mitigate these upcoming challenges generated by aging populations, modern technologies could be integrated faster in the healthcare system. Interventions designed for greater efficiency (e.g., shorter treatment durations, less personnel-intensive treatments) may result in prolonged independence for older individuals and patients, offering a prospect of alleviating the pressures on healthcare systems and health workers in the best case scenarios. In this vein, many promising technologies [e.g., telerehabilitation, wearable sensors, non-invasive brain stimulation, and virtual reality (5)] have been developed or can be seen at the horizon [e.g., vestibular implants (6)]. For example, telemedicine has been demonstrated to be an effective tool for increasing patients’ accessibility to healthcare services, for reducing healthcare costs and for improving the patient’s quality of life in the context of alcohol use disorders (7). Likewise, meta-analytic analyses on virtual reality confirmed its effectiveness in the treatment of phobias (8), in the treatment of military veterans with Post-Traumatic Stress Disorder (PTSD) (9), in the cognitive rehabilitation of patients suffering from mild cognitive impairment (MCI) (10), and in stroke rehabilitation (11). On one hand, the long list of recent technological innovations demonstrates, that engineers raised to the challenge of providing better solutions for rehabilitation and are producing these at a high pace. On the other hand, many of the reviews on technology in rehabilitation point out that, despite potentially positive outcomes (e.g., improved quality of life, effective recovery, etc.), the adoption of new technologies by stakeholders in rehabilitation practice is relatively low. For example, the rates of prosthetic usage in upper-limb amputees has been estimated around 56%, meaning that 44% of patients abandoned their prosthetic (12). Thus, despite the improvements of the technology, the clinical reality still shows a high rejection rate of cost-intensive prosthetic devices. A review on the usage of mobile technologies (e.g., wearable sensors) reported even higher rejection rates of up to 65%, and explained this, among other reasons, by a lack of technology acceptance (13).

Taken together, there is today a substantial disparity between the pace of technological innovations in rehabilitation and their tangible impact on day-to-day clinical practices. Keeping in mind the challenges of an aging population outlined above, the low adoption rates are even more concerning and make the acceptance of technology within the rehabilitation context a pivotal factor. Interventions to increase acceptance of existing (and future) technologies have the potential to improve therapy outcomes. Crucially, raising technology acceptance leads to more efficient use of existing resources. Compared to developing, scaling and commercializing yet another device, such interventions are cost-effective and rapidly deployable. The development of telemedicine in Ghana is a case in point (14, 15). This highlights interventions to increase technology acceptance as a lever for speeding up the translation of technological innovation to rehabilitation practice. However, technology acceptance has been largely overlooked in the rehabilitation context and needs to be addressed more actively.

## A possible solution: integrating the human sciences

The Technology Acceptance Model (TAM) has been developed by economists to model the acceptance and usage of computer technology by a decision maker (16). The model suggests that only two predictors, perceived usefulness and perceived ease of use, determine the intention to use a technology. More recent models, for example the Unified Theory of Acceptance and Use of Technology (UTAUT) (17), assume a larger number of predictors (performance expectancy, effort expectancy, social influence, facilitating conditions) and identified moderators (gender, age, experience, voluntariness of use). Event though TAM has been extended to the realm of rehabilitation (18) (likely due to the absence of a more suitable alternative), both models cannot be used directly to create interventions to increase technology acceptance: Both models have been developed for situations that vary significantly from a rehabilitation context. For example, both models typically assume that the decision whether or not to use a technology is made by a single decision maker and is only made once. However, within the domain of rehabilitation, a patient’s decision to adopt and use a specific technology is a complex, ongoing, and long-lasting process. Furthermore, the decision involves a multitude of stakeholders beyond patients, such as medical staff, physiotherapists, psychologists, political entities, and insurance companies. These stakeholders possess diverse preferences and, at least partially, conflicting interests. In addition, the scope of TAM is the perceived usefulness, defined as the extent to which a person believes that a technology will help them to perform their job, which is a rather restricted scope and does not translate readily to private technology use of for example elderly and retired patients.

Therefore, the available, relatively simple models of technology acceptance can not capture the rehabilitation context well enough to generate interventions that target technology acceptance. To address this issue, we propose three next steps forward:

(1) Adapting prevailing technology acceptance models to better map the intricacies of the rehabilitation context.
(2) Empirically assessing and validating existing and future technology acceptance models.
(3) Increasing the acceptance of technology among all stakeholders through the design of effective communication strategies and interventions.

To move forward in these directions, we argue that the human sciences (i.e., history, philosophy, sociology, psychology, justice studies, evolutionary biology, biochemistry, neurosciences, folklorists, and anthropology), and therein particularly psychology, have a largely untapped potential to contribute.

First, it is noteworthy that a substantial portion of the factors and moderators in existing technology acceptance models show stronger associations with human sciences, such as psychology (e.g., expectancies) and sociology (e.g., social influence), as opposed to engineering or economics. Expectations and reasoning about the potential consequences of actions (e.g., using a technology) are clearly mental cognitive processes, and therefore psychologists and neuroscientists are best trained to investigate and model them. In particular, psychology, driven by its primary mission of elucidating and predicting human behavior, has a rich body of theories and concepts that can lead to next-generation theories of technology acceptance. For example, integrating perception of agency and self-efficacy of patients can shift the focus from the patient as a passive recipient of technology, towards a more holistic understanding of patients as actors in a multi-stakeholder setting. In addition, integrating social identity theory sees the patient as a social being for which technology adoption has not only health, but also social consequences (for example, using a prosthetic makes one’s ”weakness” clearly visible, a price that not everyone might be willing to pay). Thus, psychological theories can enrich development of future models of technology acceptance.

Second, current and future models of technology acceptance need to be tested in clinical settings, for which the human sciences, and again, particularly psychology, are well-equipped with a rich methodological spectrum. Specifically, these methods allow capturing the social structures within which patients find themselves (for example, grouped in hospitals or retirement homes). This allows much more accurate inference about the underlying factors driving technology acceptance. That lack of incorporating the latest psychological advancements into decision-making processes in the medical field has extensively been criticized (19) and first attempts in this direction have already been published (20).

Third, theory development and validation must be translated to interventions and protocols that increase technology acceptance, adoption levels, and long-term usage of technology. Here again, psychology has a long track record of developing behavior change interventions based on different frameworks. On the one hand, there are interventions for changing habituated health behaviors derived from habit theory (21, 22). On the other hand, interventions can target more deliberative and seldom decisions, for example via social norms (23). For an introduction to behavior change theory and interventions, please see (24). This body of knowledge can be readily used to develop interventions for technology acceptance in the rehabilitation context.

Taken together, psychology offers the theories needed to formulate the next generation of technology acceptance models, the methodological skills to evaluate them, and the experience in designing impactful behavior change interventions. The potential of psychology is, we argue, best harnessed in inter- and transdisciplinary teams bringing together approaches from other human sciences with all stakeholders to co-create the research questions and technology development. The human sciences provide a specialized focus on human interactions, perceptions, cognitive and decision-making processes, and can look back on a successful tradition of explaining and predicting human behavior. The enhanced integration of human sciences into rehabilitation science aligns with the overarching objective of expediting technology acceptance in practical rehabilitation settings. Crucially, this can counteract the challenges posed by current and upcoming demographic shifts. Engineering sentences develop much needed new technologies, but the human sciences ensure the actual adoption of technologies and should seek and stand their important role more actively.
